# Effects of Whole-Body High-Intensity Interval Training on Cardiorespiratory Fitness, Musculoskeletal Fitness, Body Composition, and Metabolic Biomarkers in Adults with Overweight and Obesity

**DOI:** 10.3390/healthcare14172798

**Published:** 2026-09-01

**Authors:** Julie Carpentier, Bert Celie, Séverine Stragier, Vitalie Faoro, Alain Carpentier, Malgorzata Klass

**Affiliations:** Research Unit in Cardio-Respiratory Physiology, Exercise & Nutrition, Faculty of Human Movement Sciences, Université Libre de Bruxelles, 1070 Brussels, Belgium; julie.carpentier@ulb.be (J.C.); bert.celie@ulb.be (B.C.); severine.stragier@ulb.be (S.S.); vitalie.faoro@ulb.be (V.F.); alain.carpentier@ulb.be (A.C.)

**Keywords:** VO_2_peak, muscle strength, muscle endurance, adiposity, metabolic health, health-related fitness, exercise intervention, circuit training, high intensity functional training

## Abstract

**Highlights:**

**What are the main findings?**

- A 10-week whole-body high-intensity interval training (WB-HIIT) improved health-related fitness and body composition, with gains in aerobic capacity, muscular strength and endurance, and a small reduction in central adiposity.

**What are the implications of the main findings?**

- Obesity is characterized not only by metabolic impairments and reduced aerobic capacity, but also by decreased musculoskeletal fitness. Whole-body HIIT, by combining aerobic and strength components, may offer an effective approach to simultaneously stimulate the cardiovascular and neuromuscular systems.
- WB-HIIT is a time-efficient and accessible training modality that can be implemented in everyday settings, unlike traditional HIIT performed on cycle ergometers or treadmills. Its minimal equipment, whole-body nature, and adaptability may help overcome common barriers to exercise participation.

**Abstract:**

**Background/Objectives**: Identifying accessible and effective exercise interventions is essential for improving health and physical fitness in individuals with overweight or obesity. Whole-body high-intensity interval training (WB-HIIT) is a time-efficient approach that requires minimal equipment and can be adapted to a wide range of settings and fitness levels. Therefore, this study aimed to investigate the effects of WB-HIIT on cardiorespiratory and musculoskeletal fitness, body composition, and metabolic health markers. **Methods**: Thirty-six healthy adults with overweight were assigned to either a WB-HIIT group (*n* = 19; 35 ± 9 years; BMI 30.2 ± 3.1 kg/m^2^) or a control group (*n* = 17; 33 ± 11 years; BMI 30.3 ± 3.3 kg/m^2^). Participants completed pre- and post-intervention assessments including dual-energy X-ray absorptiometry (DXA), blood sampling, maximal cardiopulmonary exercise testing, and measures of muscular strength (3 RM) and functional endurance (1 min sit-to-stand and arm curl). The 10-week supervised WB-HIIT (2–3 sessions per week) included a warm-up, 3–4 sets of 10–12 all-out whole-body exercises (30–40 s) interspersed with 20–30 s recovery, and a cool-down. **Results**: The control group showed no significant changes in any of the assessed parameters after 10 weeks, except for a modest increase in body fat mass. The WB-HIIT group showed significant improvements in cardiorespiratory fitness, evidenced by increases in VO_2_peak (*p* < 0.05), maximal treadmill incline (*p* < 0.01), and first ventilatory threshold parameters (*p* < 0.05). Maximal strength increased significantly in both the leg and bench press (*p* < 0.001), accompanied by a significant improvement in upper-limb functional endurance (arm curl, *p* < 0.05). No significant changes were observed in body weight or BMI, but small reductions were found in body fat percentage, trunk fat mass, and waist circumference (*p* < 0.05). Except for a modest decrease in glycated hemoglobin (*p* < 0.05), metabolic blood parameters remained unchanged and were already within the normal range at baseline. **Conclusions**: A 10-week WB-HIIT intervention improved cardiorespiratory fitness, muscular strength, and upper-limb functional endurance, and elicited a modest reduction in trunk fat mass in individuals with overweight or obesity.

## 1. Introduction

Overweight and obesity are major global public health concerns and are strongly associated with the development of metabolic syndrome, a multifactorial condition characterized by central adiposity, insulin resistance, dyslipidemia, and chronic low-grade inflammation [1,2]. These alterations significantly increase the risk of cardiovascular diseases and type 2 diabetes, and are often accompanied by impairments in physical function and quality of life [3]. Obesity is now recognized as a complex systemic condition involving multiple physiological systems, highlighting the need for comprehensive and integrative intervention strategies [2]. In this context, physical exercise is widely acknowledged as a cornerstone in the management of overweight and obesity due to its broad effects on metabolic and functional health [4,5].

While moderate-intensity continuous training remains widely prescribed due to its safety and feasibility for individuals with low initial fitness levels, particularly those with overweight or obesity, its effectiveness for improving certain metabolic and cardiovascular outcomes may be lower than that of higher-intensity exercise modalities [6,7]. In this context, high-intensity interval training (HIIT) has emerged as a time-efficient and promising alternative. HIIT involves repeated bouts of high-intensity effort interspersed with recovery periods and has been shown to improve body composition, muscle function, and cardiorespiratory fitness in individuals with overweight and obesity [5,6,7,8,9], along with key physiological adaptations such as enhanced muscle capillarization, mitochondrial content, and insulin sensitivity that underpin obesity-related metabolic dysfunction [5].

Despite these promising findings, the current literature remains limited and fragmented, particularly regarding the specific effects of different HIIT modalities in individuals with overweight and obesity. While several studies have investigated whole-body HIIT (WB-HIIT) interventions based on multi-joint functional exercises, most have focused on isolated outcomes, such as body composition or cardiorespiratory fitness, rather than capturing integrated, multi-system adaptations [4,6,10]. In addition, many studies approach HIIT as a general concept without clearly distinguishing between exercise modalities (e.g., cycling, running, or WB-HIIT), despite their potentially distinct physiological effects [4,10]. Importantly, the majority of existing research relies on structured and equipment-based exercise modalities, which may not fully reflect real-world conditions. This limitation is particularly relevant given that accessibility and feasibility are key determinants of long-term participation.

In line with these considerations, HIIT is most commonly implemented using cyclic modalities such as cycling or running. While effective for improving metabolic and cardiorespiratory outcomes [6], these approaches primarily target aerobic capacity and may not fully address other important components of physical fitness, such as muscular strength.

Importantly, obesity is not only characterized by metabolic impairments and reduced aerobic capacity, but also by decreased musculoskeletal fitness. Current exercise recommendations therefore emphasize the combined use of aerobic and resistance training to achieve optimal health benefits in this population [11]. However, traditional HIIT modalities are often based on mono-structural exercises and typically do not incorporate a substantial strength-training component within the same session. In addition, repetitive exercise modalities may be perceived as monotonous, and the high intensities required may be challenging for individuals with low initial fitness levels, which can negatively influence adherence. Taken together, these limitations highlight the need for more adaptable and comprehensive training approaches capable of addressing multiple components of health-related fitness.

In this context, training approaches based on functional, whole-body movements may represent a relevant alternative. By integrating both aerobic and strength-related components within a single session and recruiting a larger muscle mass, these approaches simultaneously stimulate cardiovascular and neuromuscular systems. WB-HIIT specifically reflects this approach, as it relies on multi-joint functional movements performed with little or no equipment [8]. Such protocols are highly accessible and can be implemented in various settings, including at home, thereby addressing common barriers to exercise participation. Importantly, this approach offers a high degree of scalability, allowing exercise selection and intensity to be tailored to individual capabilities and limitations.

Studies using WB-HIIT protocols have demonstrated significant improvements in cardiorespiratory fitness, musculoskeletal fitness, and body composition [10,12,13,14,15]. However, most studies have not examined adaptations across multiple physiological systems simultaneously, including metabolic health, cardiorespiratory and musculoskeletal fitness, and body composition, leading to a fragmented rather than comprehensive understanding of the overall benefits. Therefore, this study aims to evaluate the simultaneous effects of a WB-HIIT intervention on cardiorespiratory and musculoskeletal fitness, body composition, and metabolic health in individuals with overweight and obesity, using a comprehensive set of outcome measures.

## 2. Materials and Methods

### 2.1. Participants

This study was designed as a controlled interventional trial aiming to investigate the effects of a WB-HIIT on body composition, metabolic health (blood sample), and cardiorespiratory and muscular adaptations in adults with overweight or obesity.

The study protocol was reviewed and approved by the Ethics Committee of Erasme–Université Libre de Bruxelles on 14 October 2022 (P2022/344, CCB B4062022000175). All procedures were conducted in accordance with the Declaration of Helsinki. Written informed consent was obtained from all participants before the beginning of the study.

Participants were recruited through advertisements on social media, newspapers, posters displayed within the Faculty of Human Movement Sciences of the Université libre de Bruxelles, as well as through word of mouth. Eligible participants included men and women aged 18–50 years with a body mass index (BMI) between 25 and 35 kg/m^2^, in whom elevated BMI was attributable to excess adiposity rather than increased lean mass, as determined by dual-energy X-ray absorptiometry (DXA). In addition, participants had no known comorbidities or chronic diseases, such as cardiovascular disease, type 2 diabetes, hypertension, or other disorders that could affect the study outcomes or limit participation in the WB-HIIT intervention, and engaged in less than 150 min of structured physical activity at moderate to vigorous intensity per week.

Based on the effect size reported in our meta-analysis investigating the effects of WB-HIIT on health-related fitness [8], an a priori sample size calculation was performed using G*Power (version 3.1.9.7; University of Düsseldorf, Germany). The calculation was based on the following parameters: two groups, two measurement time points, an effect size of 0.38, a two-tailed α level of 0.05, statistical power (1 − β) of 0.90, a non-sphericity correction of 1, and a correlation of 0.5 between repeated measures. The results indicated that a minimum total sample size of 22 participants was required to detect statistically meaningful effects.

**Figure 1:**
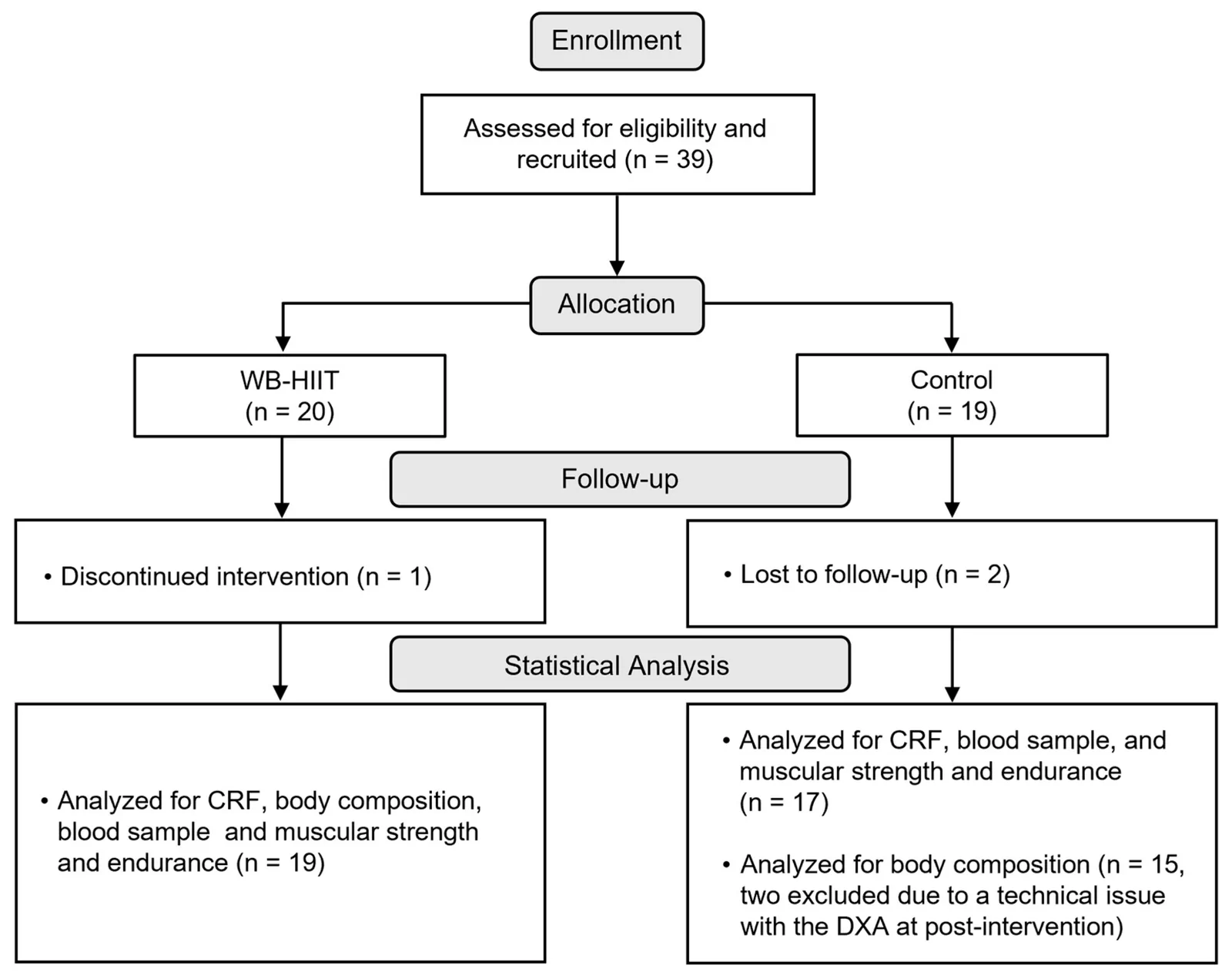
Study flowchart. CRF, cardiorespiratory fitness. DXA, dual-energy X-ray absorptiometry. WB-HIIT, whole-body high-intensity interval training.

A total of 39 participants were recruited. To ensure balanced distribution of sex between groups, participants were first stratified by sex (male and female). Block randomization was then performed within each stratum to allocate participants to the intervention group (WB-HIIT) and the control group (CTL). Twenty participants were allocated to the WB-HIIT group and 19 to the CTL group. The participant flowchart is shown in Figure 1.

In the WB-HIIT group, 1 participant discontinued the intervention. In the CTL group, two participants were lost to follow-up. The final sample comprised 19 participants in the WB-HIIT group (16 females and 3 males; age 35.0 ± 9.4 years) and 17 in the CTL group (14 females and 3 males; age 33.4 ± 11.4 years). All participants completed the post-intervention assessments, except for two participants in the CTL group who did not undergo the post-intervention DXA scan due to a technical issue with the device. Participants’ baseline characteristics are presented in Table 1.

### 2.2. Trial Registration and Transparency

The study was retrospectively registered at ClinicalTrials.gov (ID: NCT07582718), with registration completed on 13 May 2026. The reason for the retrospective registration was that the authors were not aware of the International Committee of Medical Journal Editors (ICMJE) policy requiring prospective registration of all interventional studies, including exercise interventions involving a priori healthy participants. However, the study protocol had received ethics approval (see Section 2.1) prior to participant recruitment, and no modifications were made to the study design, inclusion and exclusion criteria, or outcome measures during the conduct of the study. The trial was subsequently registered to ensure transparency and to align with current clinical trial registration and reporting standards.

### 2.3. Study Design

**Figure 2:**
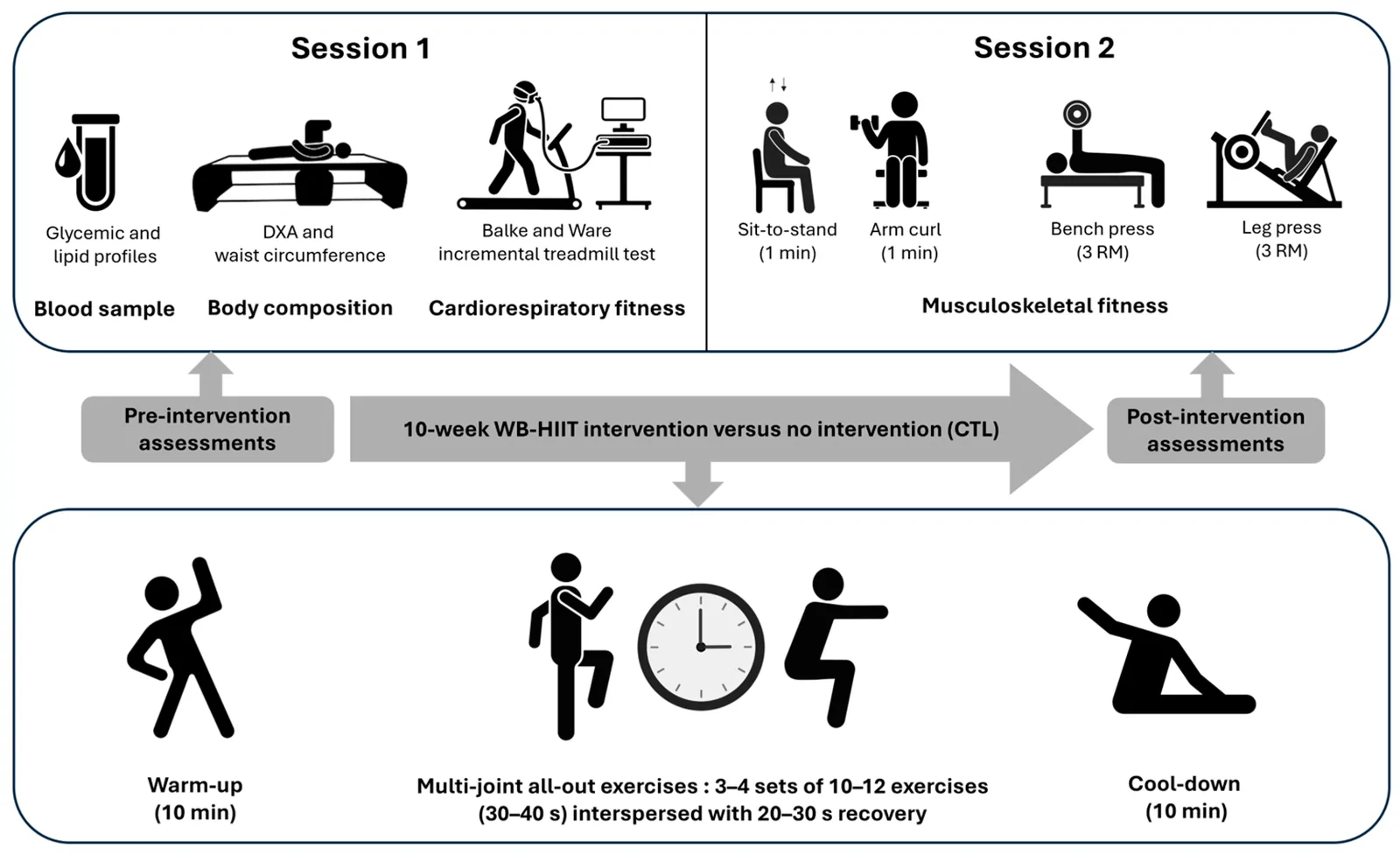
Participants took part in two assessment sessions separated by 48–72 h at pre- and post-intervention. The intervention group took part in a 10-week whole-body high-intensity interval training (WB-HIIT) consisting of a warm-up, a conditioning phase (multi-joint all-out exercises), and a cool-down. The control group (CTL) received no intervention. DXA, dual-energy X-ray absorptiometry. 3 RM, three-repetition maximum.

The study design is illustrated in Figure 2. Pre- and post-intervention evaluations were conducted over two sessions separated by 48 to 72 h. The first session included assessments of blood parameters, body composition, and cardiorespiratory fitness. The second session was dedicated to the evaluation of musculoskeletal fitness, including upper- and lower-body muscular strength and functional endurance.

### 2.4. Body Composition Assessment

All measurements were conducted under standardized conditions, with participants in a fasted state, barefoot, wearing light clothing, and having voided prior to testing.

Height, body weight, and waist circumference were assessed. Waist circumference was measured according to the World Health Organization protocol [16], at the midpoint between the lower margin of the last palpable rib and the top of the iliac crest, using a non-elastic measuring tape. Measurements were executed at the end of a normal expiration. Three consecutive measurements were obtained, and the mean value was used for analysis.

Following these assessments, total and segmental (arms, legs, and trunk) fat mass and lean mass were evaluated using DXA (Lunar Prodigy, GE HealthCare, Chicago, IL, USA) and analyzed using the enCORE software (version 15.0). Daily quality control and calibration procedures were conducted using manufacturer-provided phantoms. All DXA scans were performed in accordance with the manufacturer’s recommended positioning and scanning protocols. Participants wore light clothing, removed all metal objects, and were positioned supine with standardized limb placement during both the pre- and post-intervention assessments to ensure measurement reliability. Identical analysis procedures were applied to scans acquired at both time points.

### 2.5. Glycemic and Lipid Profiles

Fasting blood samples were collected by a certified nurse at the Erasme Medical Center following an overnight fast. Analyzed parameters included triglycerides, total cholesterol, high-density lipoprotein cholesterol (HDL-C), low-density lipoprotein cholesterol LDL-C, fasting glucose, glycated hemoglobin (HbA1c), and C-peptide.

### 2.6. Cardiorespiratory Fitness Assessment

Cardiorespiratory fitness was evaluated using the Balke and Ware incremental treadmill test [17,18] performed until volitional exhaustion during a walking protocol with progressive increases in treadmill inclination. The protocol consisted of a constant speed of 5.4 km/h, with the incline starting at 0% during the first minute, increasing to 2% after one minute, and then rising by 1% each minute thereafter [18]. This protocol is especially adapted for inactive and overweight participants thanks to its slow and gradual increase in incline and constant walking speed. Oxygen uptake (VO_2_) and carbon dioxide production were recorded breath by breath using a tightly fitted oro-nasal mask connected to a cardiopulmonary exercise system (Ergocard, Medisoft, Dinant, Belgium) calibrated with room and standardized gas. Heart rate was monitored throughout the test using a Polar^®^ H9 heart rate monitor (Polar Electro, Kempele, Finland). The test was terminated when participants were unable to maintain the required walking speed and was considered maximal when the respiratory exchange ratio reached ≥1.15. As a VO_2_ plateau was not observed in most participants, VO_2_peak was used as the indicator of maximal aerobic capacity.

Besides VO_2_peak, the cardiopulmonary exercise test enabled the determination of maximal heart rate (HRmax), the maximal treadmill incline attained at test termination, and the first ventilatory threshold (VT1), which was identified using the V-slope and ventilatory equivalent methods [19,20] by two independent investigators, with discrepancies resolved by consensus. VT1 is considered an objective marker of submaximal aerobic exercise capacity and reflects the transition from predominantly aerobic metabolism to a progressively greater contribution of anaerobic energy production [21].

### 2.7. Musculoskeletal Fitness Assessment

Functional muscular endurance was assessed using two tests: a 1 min arm curl test and a 1 min sit-to-stand test. For the arm curl test, participants were instructed to perform as many repetitions as possible within 60 s using standardized loads (2 kg for women and 4 kg for men), while maintaining proper execution throughout the movement. The sit-to-stand test was performed using a standardized 45 cm chair. Participants started from a seated position with arms crossed over the chest and were instructed to complete as many sit-to-stands as possible within 60 s. Each repetition required full extension of the lower limbs and contact with the chair during the sitting phase. For both tests, only correctly performed repetitions through a full range of motion were counted.

After a 30 min rest period, muscular strength was assessed using a three-repetition maximum (3 RM) protocol on the leg press (lower body) and bench press (upper body). Following a standardized warm-up, loads were progressively increased until participants reached failure within three repetitions, with rest periods of five minutes between attempts.

### 2.8. Exercise Intervention Protocol

During the intervention period, participants in both groups were instructed to maintain their usual dietary intake. Participants in the CTL group were also asked to maintain their habitual physical activity levels throughout the study, whereas those assigned to the intervention group completed a 10-week WB-HIIT program consisting of 24 training sessions performed 2 to 3 times per week.

The training was based on multi-joint exercises performed using minimal equipment (e.g., resistance bands and mats). Training load was progressively increased throughout the intervention period (every two weeks) by modifying one or more variables, including increasing exercise duration, reducing recovery time, increasing the number of exercises, or the number of sets. A detailed description of the WB-HIIT exercises and protocol is provided in Table 2.

Each training session was supervised and followed a structured format including a standardized warm-up, a main WB-HIIT phase, and a cool-down period. The warm-up consisted of approximately 10 min of joint mobility exercises and dynamic movements targeting the main muscle groups involved in the session. The WB-HIIT phase consisted of 3 to 4 sets of 10 to 12 multi-joint exercises performed consecutively. Each exercise was performed for 30 to 40 s, followed by a short recovery period of 20 to 30 s. Participants were instructed to complete as many repetitions as possible of each exercise (i.e., “all-out” execution) during each work interval while maintaining proper exercise technique. An additional recovery of 2.5 min was provided between sets. The session concluded with a cool-down phase consisting of stretching and breathing exercises. The total duration of the session ranged from 50 to 70 min, including warm-up and cool-down.

### 2.9. Statistical Analysis

Statistical analyses were conducted using Jamovi software (version 2.4.14). Normality of the data was assessed using the Shapiro–Wilk test and Q–Q plots. A two-factor ANOVA (intervention [WB-HIIT vs. CTL] × time [pre- vs. post-intervention]) with repeated measures on time was used to analyze training-induced changes when normality and homogeneity of variances (assessed by Levene’s test) were met. Effect sizes were reported using partial eta squared (η^2^p). Values of 0.01, 0.06, and 0.14 correspond respectively to small, medium, and large effects [22]. When a significant intervention-by-time interaction was observed, Tukey post hoc tests were performed to compare pre- to post-intervention values within each group. When ANOVA assumptions were not satisfied, within-group changes were analyzed using the nonparametric Wilcoxon test. The significance level was set at *p* < 0.05.

Pre- and post-intervention data in the WB-HITT and CTL groups are reported as mean ± standard deviation for normally distributed variables and as median [25th, 75th percentiles] for non-normally distributed variables. Between-group estimated differences in change (EMD) and their corresponding 95% confidence intervals (95% CI) are also reported in the results tables to provide an estimate of the magnitude and direction of the WB-HIIT effect relative to the CTL group. Mean differences are reported when a two-factor ANOVA was performed, whereas median differences are reported when a non-parametric test was used.

## 3. Results

**Figure 3:**
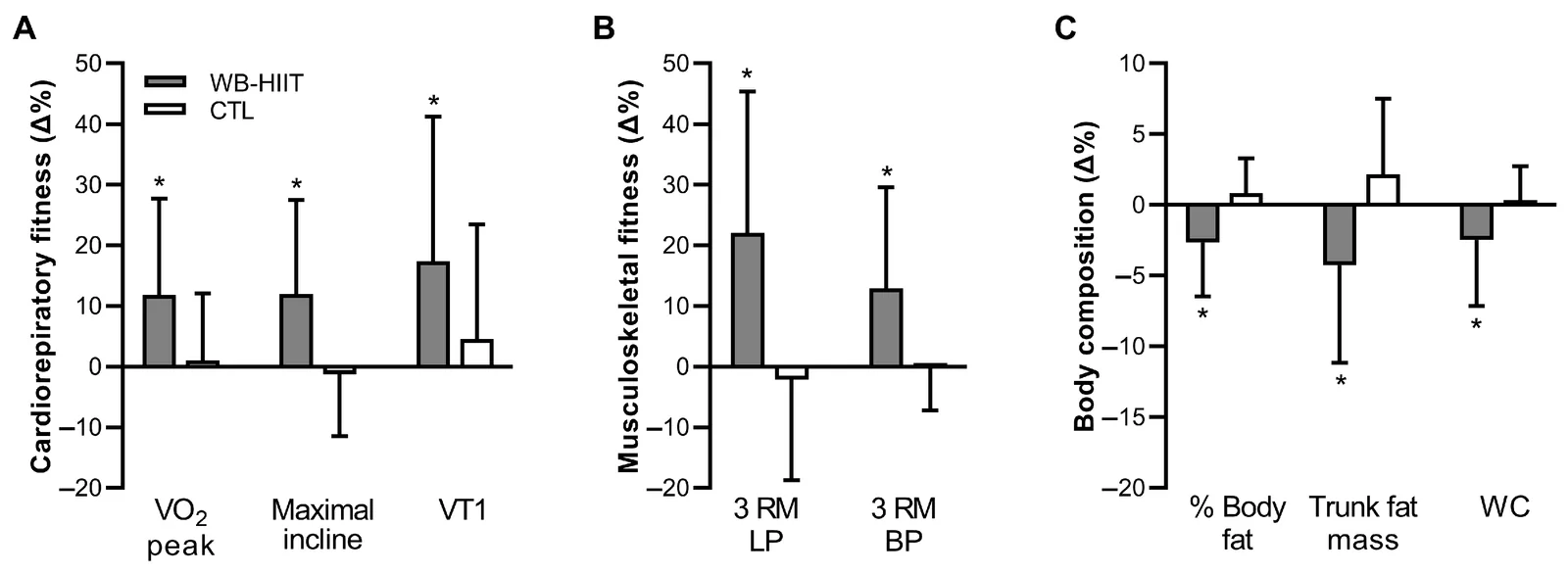
Mean percentage changes from baseline (Δ%) in cardiorespiratory fitness (**A**), musculoskeletal fitness (**B**), and body composition (**C**) outcomes following the intervention in the whole-body high-intensity interval training (WB-HIIT) and control (CTL) groups. * Significant within group change from pre- to post-intervention (*p* < 0.05). Error bars represent standard deviations. % Body fat, body fat percentage. 3 RM LP, three-repetition maximum at leg press. 3 RM BP, three-repetition maximum at bench press. VO_2_peak, peak oxygen uptake (in mL/kg/min). VT1, first ventilatory threshold (VO_2_ in mL/kg/min). WC, waist circumference.

Pre- and post-intervention values for cardiorespiratory and musculoskeletal fitness, body composition, and glycemic and lipid profiles, along with intervention effects and between-group EMDs (95% CI), are presented in the text and tables below. In addition, Figure 3 illustrates the mean percentage changes in the main outcomes from pre- to post-intervention in the WB-HIIT and CTL groups, providing a visual overview of the intervention effects.

### 3.1. Cardiorespiratory Fitness

The ANOVA analysis revealed a significant intervention by time interaction for VO_2_peak (absolute and relative to body and lean mass) and maximal treadmill incline, indicating a different time course for these parameters between the WB-HIIT and CTL groups, while no significant interaction was found for HRmax (*p*-values in Table 3). More specifically, Tukey post hoc analyses showed increases in absolute VO_2_peak (*p* = 0.008), VO_2_peak relative to body and lean mass (*p* = 0.004 and 0.028, respectively), and maximal incline (*p* = 0.004) after WB-HIIT, whereas no significant changes occurred in the CTL group (*p*-values range: 0.756–0.99; Figure 3A).

As VT1 variables were not normally distributed, within-group pre- to post-intervention changes were assessed using Wilcoxon tests. Significant improvements were observed in the WB-HIIT group (*p*-values range: 0.005–0.040), whereas no significant changes were found in the CTL group for absolute and relative VO_2_ or incline measured at VT1 (*p*-values range: 0.282–0.720; Figure 3A).

### 3.2. Musculoskeletal Fitness

The ANOVA analysis revealed a significant intervention by time interaction for 3 RM at the leg press and bench press (*p*-values in Table 4). Tukey post hoc analyses showed increases in leg press and bench press 3 RM following WB-HIIT (both *p* <0.001; Figure 3B), whereas no significant changes were observed in the CTL group (*p* = 0.866 and 0.999, respectively). For the 1 min arm curl and sit-to-stand tests, Wilcoxon analyses showed a significant increase in arm curl performance (*p* = 0.009) following WB-HIIT, whereas the improvement in sit-to-stand performance did not reach statistical significance (*p* = 0.074). No significant changes were observed in the CTL group for the 1 min arm curl and sit-to-stand tests (*p* = 0.395 and 0.877, respectively).

### 3.3. Body Composition

Regarding the anthropometric measures, the ANOVA analysis revealed a significant intervention by time interaction for waist circumference, but not for body weight or BMI (*p*-values in Table 5). Tukey post hoc analyses indicated that waist circumference decreased following WB-HIIT, whereas it remained unchanged in the CTL group (*p* = 0.013 and 0.951, respectively; Figure 3C).

Regarding total and segmental body composition, the ANOVA analysis revealed significant intervention by time interactions for body fat percentage and trunk fat mass (kg), whereas no significant interactions were observed for the other parameters (*p*-values in Table 5). Tukey post hoc analyses confirmed small but significant reductions in body fat percentage and trunk fat mass (kg) following WB-HIIT (*p* = 0.015 and 0.027, respectively), while no significant changes were observed in the CTL group (*p* = 0.769 and 0.393, respectively; Figure 3C).

Due to non-normal data distribution, total fat mass (kg) was analyzed using Wilcoxon tests within each group. No significant change was observed in the WB-HIIT group (*p* = 0.080), whereas the CTL group showed a small but significant increase post-intervention compared with baseline (*p* = 0.015).

### 3.4. Glycemic and Lipid Profiles

The ANOVA did not reveal any significant interaction between intervention and time for cholesterol, HDL-C, and C-peptide (*p*-values in Table 6). For parameters violating the normality assumption (triglycerides, LDL-C, fasting glucose, and HbA1c), Wilcoxon tests did not reveal any significant change from pre- to post-intervention (*p*-values range: 0.178–0.587 in WB-HIIT and 0.234–0.868 in CTL), except for HbA1c, which exhibited a modest but significant decrease in the WB-HIIT group (*p* = 0.026).

## 4. Discussion

The aim of the present study was to evaluate the effects of a 10-week WB-HIIT intervention on cardiorespiratory fitness, musculoskeletal fitness, body composition, and metabolic health in individuals with overweight and obesity. The main findings indicate that WB-HIIT significantly improved cardiorespiratory fitness and musculoskeletal fitness, and led to a small reduction in body fat percentage, trunk fat mass (kg), and waist circumference, while metabolic blood markers remained largely stable. These findings support the ability of the WB-HIIT intervention to simultaneously stimulate the cardiorespiratory and neuromuscular systems, highlighting its potential role in the management of overweight and obesity, which are associated with impairments across multiple components of health-related fitness [11].

### 4.1. Cardiorespiratory Fitness

WB-HIIT induced significant improvements in VO_2_ at VT1, VO_2_peak, and maximal treadmill incline reached at VT1 and at the end of the test. These findings indicate improvements in both submaximal and maximal aerobic capacity, which are key determinants of functional capacity and cardiovascular health in individuals with overweight and obesity [4]. The increase in VO_2_peak (~12%) is consistent with the magnitude of improvements reported in previous WB-HIIT interventions of similar duration. Indeed, most studies conducted over 8–12 weeks report increases in VO_2_peak ranging from 7 to 15%, measured either by direct laboratory assessment or by indirect field-based methods, in individuals with overweight and obesity [10,13,15,23].

In the present study, aerobic capacity was assessed using direct cardiopulmonary exercise testing, considered the gold standard, which likely provides a more accurate quantification of training-induced changes compared to indirect approaches, and also allows monitoring of changes in VO_2_ at VT1. The improvement in VT1 is particularly relevant, as it reflects an enhanced ability to sustain prolonged submaximal exercise and delay the onset of anaerobic metabolism [19,21,24]. The concurrent increase in VO_2_peak and VO_2_ at VT1 suggests that both central and peripheral adaptations occurred, potentially including improved oxygen delivery and muscle aerobic capacity [5,25,26]. These adaptations are consistent with previous findings demonstrating that HIIT can induce rapid improvements in aerobic capacity, even with reduced training volume [4,10].

### 4.2. Musculoskeletal Fitness

In addition to cardiorespiratory adaptations, WB-HIIT led to significant improvements in maximal muscular strength and functional endurance. These findings are consistent with previous studies showing that WB-HIIT protocols can enhance neuromuscular performance in individuals with overweight and obesity [8,13,15]. The observed improvements in maximal muscular strength, along with task-specific improvements in upper-limb functional muscular endurance, likely reflect favorable muscular and neural adaptations [26].

From a broader perspective, these findings support the concept that WB-HIIT acts as a multimodal stimulus capable of simultaneously targeting cardiovascular and neuromuscular systems [26]. Such an approach is particularly relevant in individuals with overweight and obesity, who often present impairments across multiple physiological domains. From a clinical perspective, the neuromuscular adaptations are particularly relevant, as skeletal muscle plays a central role in metabolic regulation through glucose uptake and endocrine signaling via myokine release [5]. Therefore, improvements in muscle function may contribute to metabolic health independently of changes in body composition.

### 4.3. Body Composition

No significant changes were observed in total body weight or BMI, while small but significant reductions in waist circumference, body fat percentage, and trunk fat mass (2.5 to 4%) were identified following the intervention. These findings suggest that WB-HIIT may preferentially target central adiposity, which is strongly associated with increased cardiometabolic risk [2].

Interestingly, the absence of significant changes in body weight despite reductions in trunk fat mass suggests the occurrence of favorable body composition, whereby decreases in adiposity are not necessarily accompanied by a reduction in total body weight and BMI. This phenomenon is commonly observed in exercise interventions without dietary restriction and is consistent with previous studies indicating that HIIT can induce favorable changes in body composition without necessarily resulting in significant weight loss [6,9]. These results highlight the importance of assessing regional fat distribution and body composition rather than relying solely on body weight and BMI as indicators of health improvement. They are also consistent with previous findings emphasizing the need to look beyond BMI when assessing health risks in young adults [27].

### 4.4. Glycemic and Lipid Profiles

Despite these favorable adaptations in physical fitness and body composition, no significant changes were observed in most glycemic and lipid parameters, except for a small reduction in HbA1c. The improvement in HbA1c without a concurrent reduction in fasting glucose may be explained by the fact that the latter reflects an acute measurement, capturing glucose levels at a single time point, whereas HbA1c represents an average of glycemic changes over time, and is therefore considered a more reliable indicator of overall glycemic control [28]. The modest improvement in HbA1c observed in the present study (EMD = −0.20%) was smaller than the reductions reported in the meta-analyses by Lu et al. [29] (EMD = −0.70%) and Jelleyman et al. [30] (EMD = −0.47%) following various HIIT interventions in individuals with overweight or obesity and prediabetes or type 2 diabetes. Therefore, the clinical relevance of the present finding should be interpreted with caution.

Although these results may appear inconsistent with studies reporting greater improvements in metabolic markers following HIIT interventions [23], they are in line with recent meta-analyses showing limited or inconsistent effects of HIIT on glycemic control and lipid profiles in individuals with relatively normal baseline metabolic values, as observed in the present study [4,10,30]. Indeed, individuals with overweight but without marked metabolic dysfunction generally exhibit a lower potential for improvement in glycemic and lipid biomarkers following exercise interventions. This interpretation is supported by the concept of “metabolically healthy obesity” [31], whereby individuals, despite excess adiposity, display relatively preserved cardiometabolic health and therefore a reduced potential for measurable improvements in traditional metabolic risk markers [4].

In addition to baseline metabolic status, other participant characteristics may also have contributed to the near absence of measurable metabolic changes. For instance, Hosseini Moshkenani et al. [23] reported reductions in fasting glucose and triglycerides following WB-HIIT in a sample composed exclusively of men, whereas the present study included predominantly women and only a few men. This difference may be relevant, as premenopausal women are known to benefit from protective effects of estrogen on insulin sensitivity and lipid metabolism [32]. Furthermore, the relatively short duration of the intervention may have been insufficient to induce systemic metabolic adaptations, which often require longer-term exposure or combined interventions including dietary modifications [8,29]. This suggests that while WB-HIIT is effective in improving physical fitness and body composition, its impact on metabolic biomarkers depends largely on baseline metabolic status, as well as intervention duration, sex-related factors, and associated lifestyle conditions.

### 4.5. Health-Related and Practical Implications

From a practical perspective, WB-HIIT represents a time-efficient and highly accessible training modality that can be easily implemented in real-world settings, in contrast to more traditional HIIT protocols typically performed using cycle ergometers or treadmills. Its low equipment requirements and functional, whole-body nature may help overcome common barriers to exercise participation, such as lack of time, access to facilities, or limited exercise experience [11].

In addition to reductions in abdominal adiposity, as reflected by decreases in trunk fat mass and waist circumference, which lower cardiovascular risks [33], the 10-week WB-HIIT intervention produced significant improvements in physical fitness. Aerobic capacity increased, as demonstrated by higher VO_2_ at VT1, VO_2_peak, as well as greater treadmill incline achieved at both VT1 and maximal effort. Furthermore, participants showed improvements in lower- and upper-body muscular strength, as assessed by 3 RM tests, as well as enhanced upper-limb muscular endurance, measured by the 1 min arm curl test. These findings highlight the potential of a relatively short WB-HIIT intervention to simultaneously enhance multiple domains of health-related fitness and support its use as a comprehensive and ecologically valid intervention strategy for individuals with overweight and obesity, who often exhibit impairments across several physiological systems [2,4].

Cardiorespiratory fitness is a strong and independent predictor of health outcomes, and each 1-MET increase in fitness level has been associated with an approximately 14% lower risk of all-cause mortality [34,35]. Given that 1 MET has been estimated at 2.6 ± 0.4 mL O_2_/kg/min in a large heterogeneous sample that included individuals with overweight and obesity [36], the ~12% increase in VO_2_peak observed in the present study (EMD = 2.9 mL O_2_/min/kg) represents a clinically meaningful improvement in cardiorespiratory fitness. The concomitant enhancement in musculoskeletal fitness is also noteworthy, as greater muscular strength is independently associated with lower all-cause mortality and better functional capacity [37].

In contrast, almost no improvements were observed in glycemic or lipid blood parameters among our participants with normal baseline values. Nevertheless, growing evidence suggests that metabolically healthy obesity is not entirely benign, as individuals may still exhibit abnormalities in fat distribution, adiponectin signaling, chronic inflammation, oxidative stress, and other molecular-level (omics) markers despite having normal metabolism in the criteria of metabolic syndrome [31]. By reducing abdominal fat, oxidative stress, and chronic inflammation, and by improving adiponectin levels and metabolic function, exercise constitutes an important strategy for lowering the health risks associated with metabolically healthy obesity [31].

These results highlight the potential of WB-HIIT as a practical and effective approach for improving physical fitness and body composition, while potentially promoting metabolic health in individuals with overweight and obesity, especially in contexts where access to equipment or structured exercise facilities is limited.

### 4.6. Limitations and Perspectives

The present study has several strengths that should be acknowledged. First, cardiorespiratory fitness was assessed using direct cardiopulmonary exercise testing, which is considered the gold standard for evaluating aerobic capacity and provides a more accurate and sensitive assessment of training-induced adaptations compared to indirect estimation methods. Second, body composition was measured using DXA, allowing a precise evaluation of total and regional fat mass and lean mass, which strengthens the interpretation of the observed changes.

However, some limitations should also be considered. Given the nature of the intervention and study design, neither participants nor investigators were blinded to group allocation. This may have introduced expectancy or motivational effects that could have influenced some outcomes. However, assessments were standardized and conducted using objective physiological and performance measures, which likely reduced the risk of measurement bias. Another limitation is the use of a passive rather than an active CTL group. However, the primary aim of this study was not to compare the effectiveness of different exercise interventions, but to determine whether WB-HIIT could elicit concurrent adaptations in metabolic health, cardiorespiratory fitness, musculoskeletal fitness, and body composition in adults with overweight or obesity. Although improvements may be expected with exercise compared with no exercise, the magnitude of adaptations across these physiological domains cannot be assumed a priori, particularly given the limited evidence on WB-HIIT in this population. A major limitation of this study is that participants exhibited relatively normal baseline metabolic profiles, which may have limited the potential for detecting significant improvements in glycemic and lipid parameters. In addition, the duration of the intervention may not have been sufficient to induce measurable systemic metabolic adaptations, which may require longer-term exposure or combined lifestyle interventions, including dietary modifications. Furthermore, the broad age range of the study population and the non-collection of comprehensive sociodemographic and lifestyle data, which could have contributed to variability in the responses to the intervention, represent additional limitations of the study. The lack of a detailed assessment of neural and muscle-specific adaptations also limits the interpretation of the mechanisms underlying the observed effects. Therefore, the precise physiological mechanisms underpinning the observed responses remain to be fully elucidated. Finally, our sample consisted predominantly of women, which may limit the generalizability of the findings to men. Sex-specific differences in physiological responses to exercise may influence the observed adaptations, and therefore warrant further investigation.

Future research should aim to investigate the effects of WB-HIIT in populations with impaired metabolic profiles, where greater improvements in metabolic markers may be expected. In addition, studies combining WB-HIIT with nutritional interventions could help to better understand the interaction between exercise and diet on metabolic health. Finally, further research exploring neural and intramuscular adaptations, as well as sex differences, would provide valuable insights into the mechanisms underlying the benefits of WB-HIIT in individuals with overweight and obesity.

## 5. Conclusions

In conclusion, a 10-week WB-HIIT intervention improved cardiorespiratory fitness, musculoskeletal fitness, and abdominal adiposity in individuals with overweight and obesity. Although metabolic biomarkers remained largely unchanged in this metabolically healthy cohort, these findings highlight WB-HIIT as an effective strategy for improving health-related fitness and body composition, and for supporting long-term cardiometabolic health. Given its functional and equipment-free nature, WB-HIIT represents a promising alternative to traditional exercise approaches, with strong potential for implementation in various settings.

## Figures and Tables

**Table 1:** Participants’ characteristics in WB-HIIT and CTL groups.

Parameter	WB-HIIT	CTL
Number of subjects (female/male)	19 (16/3)	17 (14/3)
Age (years)	35.0 ± 9.4	33.4 ± 11.4
Height (cm)	169.1 ± 6.6	169.3 ± 7.9
Body weight (kg)	86.4 ± 10.4	86.8 ± 10.3
BMI (kg/m^2^)	30.2 ± 3.1	30.3 ± 3.3
Moderate to vigorous physical activity (min)	39.4 ± 40.6	62.9 ± 44.3

BMI, body mass index. CTL, control group. WB-HIIT, whole-body high-intensity interval training group.

**Table 2:** Description of the whole-body high-intensity intervention exercises and protocol.

Exercise Number	Weeks 1–2	Weeks 3–4	Weeks 5–6	Weeks 7–8	Weeks 9–10
1	Front lunges	Lateral lunges	Squat Jumps	Squat jumps	Jumping lunges
2	Front plank	Mountain climbers	Mountain climbers	Mountain climbers	Front plank
3	Squats	Squats	Squats	Front lunges	Squat jumps
4	Dips	Dips	Dips	Dips	Mountain climbers
5	Jumping jacks	Rope skipping	Rope skipping	Jumping jacks	Jumping jacks
6	Hip thrusts	Hip thrusts	Crunches	Biceps curls *	Dips
7	Burpees ^†^	Biceps curls *	Biceps curls *	Burpees	Rope skipping
8	Bird dogs	Burpees ^†^	Burpees	Bird dogs	Hip thrusts
9	Push-ups	Bird dogs	Push-ups	Push-ups	Arm curls
10	Knee elevations	Push-ups	Knee elevations	Knee elevations	Burpees
11	-	Knee elevations	-	Front plank	Bird dogs
12	-		-	-	Knee elevations
Number of sets	3 sets	3 sets	4 sets	4 sets	4 sets
Number of exercises per set	10	11	10	11	12
Exercise to rest ratio (s)	30/30	30/20	30/20	35/20	40/20
Rest between sets (min)	2.5	2.5	2.5	2.5	2.5

* Biceps curls with resistance band. ^†^ Low-impact burpees.

**Table 3:** Pre- to post-intervention comparison of the maximal cardiopulmonary exercise test parameters.

Parameter	Pre	Post	ANOVA Time × Group *p*-Value, η^2^p	EMD (95% CI)
VO_2_peak (L/min)				
WB-HIIT	2.25 ± 0.44	2.47 ± 0.42 *	*p* = 0.028, η^2^p = 0.135	0.22 (0.03 to 0.41)
CTL	2.40 ± 0.56	2.41 ± 0.40		
VO_2_peak/BW (mL/kg/min)				
WB-HIIT	26.1 ± 4.1	28.9 ± 4.7 *	*p* = 0.012, η^2^p = 0.170	2.9 (0.7 to 5.2)
CTL	27.5 ± 4.9	27.5 ± 3.4		
VO_2_peak/LM (mL/kg/min)				
WB-HIIT	47.9 ± 5.8	51.9 ± 5.3 *	*p* = 0.046, η^2^p = 0.118	4.2 (0.3 to 8.1)
CTL	49.4 ± 5.7	49.2 ± 3.9		
Maximal Incline (°)				
WB-HIIT	11.9 ± 2.3	13.2 ± 2.4 *	*p* = 0.002, η^2^p = 0.242	1.6 (0.6 to 2.6)
CTL	12.5 ± 2.9	12.2 ± 2.3		
HRmax (bpm)				
WB-HIIT	182 ± 12	183 ± 11	*p* = 0.214, η^2^p = 0.045	2 (1 to 6)
CTL	181 ± 13	179 ± 14		
VO_2_ at VT1 (L/min)				
WB-HIIT	1.42 [1.31, 1.69]	1.73 [1.52,1.98] *	N/A	0.20 (−0.08 to 0.48) ^$^
CTL	1.26 [1.12, 1.62]	1.44 [1.12,1.69]		
VO_2_ at VT1 (mL/kg/min)				
WB-HIIT	17.2 [15.5, 19.9]	21.4 [16.7, 23.7] *	N/A	3.7 (0.9 to 6.6) ^$^
CTL	15.5 [12.9, 19.6]	15.1 [13.5,19.5]		
VO_2_ at VT1/LM (mL/kg/min)				
WB-HIIT	31.5 [29.0, 34.7]	39.5 [31.0, 42.1] *	N/A	4.9 (−0.8 to 10.5) ^$^
CTL	28.4 [24.3, 32.1]	27.1 [23.5, 33.4]		
Incline at VT1 (°)				
WB-HIIT	5.0 [3.5, 5.0]	6.0 [4.5, 8.5] *	N/A	1.0 (−0.9 to 2.9) ^$^
CTL	3.0 [2.0, 5.0]	3.0 [3.0, 5.0]		

Data presented as mean ± standard deviation or median [25th, 75th percentile]. * Significantly different from pre-intervention value within this group (*p* < 0.05). BW, body weight. CTL, control group. EMD (95% CI), estimated between-group difference in change and corresponding 95% confidence interval. Mean differences are reported unless indicated by ^$^, in which case median differences are reported when a non-parametric test was performed. HRmax, maximal heart rate. Incline, incline of the treadmill. LM, total lean mass. N/A, not applicable. η^2^p, partial eta squared. VO_2_ at VT1, oxygen uptake at VT1. VO_2_peak, peak oxygen uptake. VT1, first ventilatory threshold. WB-HIIT, whole-body high-intensity interval training group.

**Table 4:** Pre- to post-intervention comparison of the parameters related to musculoskeletal fitness.

Parameter	Pre	Post	ANOVA Time × Group *p*-Value, η^2^p	EMD (95% CI)
3 RM—Leg Press (kg)				
WB-HIIT	226 ± 64	268 ± 58 *	*p* = 0.001, η^2^p = 0.272	49 (21 to 78)
CTL	246 ± 60	238 ± 61		
3 RM—Bench Press (kg)				
WB-HIIT	33 ± 10	37 ± 12 *	*p* = 0.003, η^2^p = 0.204	4 (1 to 7)
CTL	39 ± 14	39 ± 15		
1 min Sit-to-stand test (rep)				
WB-HIIT	36 [32, 47]	43 [39, 47]	N/A	3 (−3 to 9) ^$^
CTL	42 [35, 49]	44 [33, 49]		
1 min Arm curl test (rep)				
WB-HIIT	45 [40, 52]	53 [47, 60] *	N/A	5 (−3 to 13) ^$^
CTL	46 [32, 55]	42 [40, 51]		

Data presented as mean ± standard deviation or median [25th, 75th percentile]. * Significantly different from pre-intervention value within this group (*p* < 0.05). CTL, control group. EMD (95% CI), estimated between-group difference in change and corresponding 95% confidence interval. Mean differences are reported unless indicated by ^$^, in which case median differences are reported when a non-parametric test was performed. N/A, not applicable. η^2^p, partial eta squared. 3 RM, three-repetition maximum. WB-HIIT, whole-body high-intensity interval training group.

**Table 5:** Pre- to post-intervention comparison of anthropometric and body composition parameters.

Parameter	Pre	Post	ANOVA Time × Group *p*-Value, η^2^p	EMD (95% CI)
Body Weight (kg)				
WB-HIIT	86.4 ± 10.4	86.1 ± 10.9	*p* = 0.086, η^2^p = 0.084	−1.3 (−2.7 to 0.2)
CTL	86.8 ± 10.3	87.8 ± 10.9		
BMI (kg/m^2^)				
WB-HIIT	30.2 ± 3.1	30.1 ± 3.4	*p* = 0.280, η^2^p = 0.034	−0.3 (−0.8 to 0.3)
CTL	30.3 ± 3.3	30.5 ± 3.1		
Waist circumference (cm)				
WB-HIIT	98.4 ± 9.9	95.7 ± 8.8 *	*p* = 0.023, η^2^p = 0.142	−2.9 (−5.4 to −0.4)
CTL	98.0 ± 9.7	98.3 ± 9.3		
Body fat percentage (%)				
WB-HIIT	42.6 ± 5.2	41.5 ± 5.8 *	*p* = 0.002, η^2^p = 0.253	−1.3 (−2.2 to −0.5)
CTL	39.9 ± 5.3	40.2 ± 5.2		
Body fat mass (kg)				
WB-HIIT	34.6 [31.7, 44.2]	34.2 [30.9, 45.0]	N/A	−2.1 (−3.3 to −0.8) ^$^
CTL	32.1 [28.9, 40.3]	33.2 [29.8, 40.8] *		
Leg lean mass (kg)				
WB-HIIT	18.7 ± 1.8	19.2 ± 1.8	*p* = 0.337, η^2^p = 0.029	0.3 (−0.3 to 0.8)
CTL	20.2 ± 3.3	20.4 ± 3.3		
Arm fat mass (kg)				
WB-HIIT	4.0 ± 0.7	3.9 ± 0.9	*p* = 0.061, η^2^p = 0.106	−0.2 (−0.5 to 0.0)
CTL	3.6 ± 1.0	3.7 ± 0.9		
Arm lean mass (kg)				
WB-HIIT	5.1 ± 0.9	5.1 ± 0.9	*p* = 0.471, η^2^p = 0.016	0.1 (−0.2 to 0.4)
CTL	5.6 ± 1.2	5.6 ± 1.2		
Trunk fat mass (kg)				
WB-HIIT	16.9 ± 4.3	16.2 ± 4.5 *	*p* = 0.003, η^2^p = 0.239	−1.1 (−1.8 to −0.4)
CTL	16.6 ± 4.9	17.0 ± 5.4		
Trunk lean mass (kg)				
WB-HIIT	19.9 ± 2.5	19.9 ± 2.6	*p* = 0.547, η^2^p = 0.011	−0.3 (−1.1 to 0.6)
CTL	20.7 ± 2.7	21.0 ± 3.0		

Data presented as mean ± standard deviation or median [25th, 75th percentile]. * Significantly different from pre-intervention value within this group (*p* < 0.05). BMI, body mass index. CTL, control group. EMD (95% CI), estimated between-group difference in change and corresponding 95% confidence interval. Mean differences are reported unless indicated by ^$^, in which case median differences are reported when a non-parametric test was performed. N/A, not applicable. η^2^p, partial eta squared. WB-HIIT, whole-body high-intensity interval training group.

**Table 6:** Pre- to post-intervention comparison of blood metabolic parameters.

Parameter	Pre	Post	ANOVA Time × Group *p*-Value, η^2^p	EMD (95% CI)
Triglycerides (mg/dL)				
WB-HIIT	78.0 [59.0, 117.0]	80.0 [65.0 108.0]	N/A	7.0 (−19.5 to 33.5) ^$^
CTL	97.0 [74.0, 158.0]	105.0 [75.0, 140.0]		
Cholesterol (mg/dL)				
WB-HIIT	187.0 ± 33.5	188.0 ± 31.1	*p* = 0.945, η^2^p = 0.000	−0.5 (−14.6 to 13.7)
CTL	181.0 ± 30.4	182.0 ± 44.8		
HDL-C (mg/dL)				
WB-HIIT	61.3 ± 20.5	63.4 ± 22.2	*p* = 0.875, η^2^p = 0.001	−0.4 (−5.0 to 4.2)
CTL	50.9 ± 11.7	53.3 ± 13.00		
LDL-C (mg/dL)				
WB-HIIT	108.0 [87.5, 120.0]	104.0 [92.0 111.0]	N/A	−9.0 (−24.3 to 6.3) ^$^
CTL	102 [83.0,128.0]	98 [86.0, 118.0]		
Fasting glucose (mg/dL)				
WB-HIIT	88.0 [83.5, 101.0]	89.0 [86.0, 93.0]	N/A	−1.0 (−7.9 to 5.9) ^$^
CTL	87.0 [81.0, 94.0]	91.0 [83.0, 94.0]		
HbA1c (%)				
WB-HIIT	5.3 [5.1, 5.5]	5.2 [5.0, 5.4] *	N/A	−0.2 (−0.4 to 0.0) ^$^
CTL	5.4 [5.2, 5.5]	5.3 [5.1, 5.6]		
C-peptide (nmol/L)				
WB-HIIT	0.81 ± 0.22	0.80 ± 0.23	*p* = 0.432, η^2^p = 0.019	−0.1 (−0.2 to 0.1)
CTL	0.91 ± 0.25	0.95 ± 0.34		

Data presented as mean ± standard deviation or median [25th, 75th percentile]. * Significantly different from pre-intervention value within this group (*p* < 0.05). CTL, control group. EMD (95% CI), estimated between-group difference in change and corresponding 95% confidence interval. Mean differences are reported unless indicated by ^$^, in which case median differences are reported when a non-parametric test was performed. HbA1c, glycated hemoglobin. HDL-C, high-density lipoprotein cholesterol. LDL-C, low-density lipoprotein cholesterol. N/A, not applicable. η^2^p, partial eta squared. WB-HIIT, whole-body high intensity interval training group. In the CTL group, HbA1c data were unavailable for 1 participant and C-peptide data for 2 participants, as these analyses were not provided in the laboratory report.

## Data Availability

The raw data supporting the conclusions of this article will be made available by the authors upon request. The data are not publicly available due to ethical and privacy restrictions.

## Abbreviations

The following abbreviations are used in this manuscript:

BMI	Body Mass Index
BW	Body Weight
CI	Confidence interval
CRF	Cardiorespiratory Fitness
CTL	Control
DXA	Dual-Energy X-ray Absorptiometry
EMD	Estimated mean/median difference
HbA1c	Glycated Hemoglobin
HDL-C	High-Density Lipoprotein Cholesterol
HIIT	High-Intensity Interval Training
HRmax	Maximal Heart Rate
LDL-C	Low-Density Lipoprotein Cholesterol
LM	Lean Mass
η^2^p	partial eta squared
3 RM	Three-repetition maximum
VO_2_	Oxygen Uptake
VO_2_peak	Peak Oxygen Uptake
VT1	First Ventilatory Threshold
WB-HIIT	Whole-Body High-Intensity Interval Training

## References

[1] Braggio M., Dorelli G., Olivato N., Lamberti V., Valenti M., Dalle Carbonare L., Cominacini M. (2025). Tailored Exercise Intervention in Metabolic Syndrome: Cardiometabolic Improvements Beyond Weight Loss and Diet—A Prospective Observational Study. Nutrients.

[2] Srikanthan K., Feyh A., Visweshwar H., Shapiro J.I., Sodhi K. (2016). Systematic Review of Metabolic Syndrome Biomarkers: A Panel for Early Detection, Management, and Risk Stratification in the West Virginian Population. Int. J. Med. Sci..

[3] Obesity and Overweight. https://www.who.int/news-room/fact-sheets/detail/obesity-and-overweight.

[4] Wang H., Cheng R., Xie L., Hu F. (2024). Comparative efficacy of exercise training modes on systemic metabolic health in adults with overweight and obesity: A network meta-analysis of randomized controlled trials. Front. Endocrinol..

[5] Scott S.N., Shepherd S.O., Hopkins N., Dawson E.A., Strauss J.A., Wright D.J., Cooper R.G., Kumar P., Wagenmakers A.J.M., Cocks M. (2019). Home-hit improves muscle capillarisation and eNOS/NAD(P)Hoxidase protein ratio in obese individuals with elevated cardiovascular disease risk. J. Physiol..

[6] Sanca-Valeriano S., Espinola-Sánchez M., Caballero-Alvarado J., Canelo-Aybar C. (2023). Effect of high-intensity interval training compared to moderate-intensity continuous training on body composition and insulin sensitivity in overweight and obese adults: A systematic review and meta-analysis. Heliyon.

[7] Shishira K.B., Vaishali K., Kadavigere R., Sukumar S., Shivashankara K.N., Pullinger S.A., Bommasamudram T. (2024). Effects of high-intensity interval training versus moderate-intensity continuous training on vascular function among individuals with overweight and obesity—A systematic review. Int. J. Obes..

[8] Scoubeau C., Bonnechère B., Cnop M., Faoro V., Klass M. (2022). Effectiveness of Whole-Body High-Intensity Interval Training on Health-Related Fitness: A Systematic Review and Meta-Analysis. Int. J. Environ. Res. Public Health.

[9] Gaweł E., Hall B., Siatkowski S., Grabowska A., Zwierzchowska A. (2024). The Combined Effects of High-Intensity Interval Exercise Training and Dietary Supplementation on Reduction of Body Fat in Adults with Overweight and Obesity: A Systematic Review. Nutrients.

[10] Cao M., Yang B., Tang Y., Wang C., Yin L. (2024). Effects of low-volume functional and running high-intensity interval training on physical fitness in young adults with overweight/obesity. Front. Physiol..

[11] Oppert J., Bellicha A., Van Baak M.A., Battista F., Beaulieu K., Blundell J.E., Carraça E.V., Encantado J., Ermolao A., Pramono A. (2021). Exercise training in the management of overweight and obesity in adults: Synthesis of the evidence and recommendations from the European Association for the Study of Obesity Physical Activity Working Group. Obes. Rev..

[12] Batrakoulis A., Jamurtas A.Z., Georgakouli K., Draganidis D., Deli C.K., Papanikolaou K., Avloniti A., Chatzinikolaou A., Leontsini D., Tsimeas P. (2018). High intensity, circuit-type integrated neuromuscular training alters energy balance and reduces body mass and fat in obese women: A 10-month training-detraining randomized controlled trial. PLoS ONE.

[13] Irene-Chrysovalanto T., Petros A., Manos S. (2022). Effects of High-Intensity Circuit Training in Obese and Overweight Population: A Randomized Clinical Trial. Int. J. Sports Exerc. Med..

[14] Mohr M., Rago V. (2023). Full-Body Circuit Training Improves Body Composition and Cardiorespiratory Fitness in Overweight Sedentary Adults: A Randomized Controlled Trial. Coll. Antropol..

[15] Sperlich B., Wallmann-Sperlich B., Zinner C., Von Stauffenberg V., Losert H., Holmberg H.C. (2017). Functional High-Intensity Circuit Training Improves Body Composition, Peak Oxygen Uptake, Strength, and Alters Certain Dimensions of Quality of Life in Overweight Women. Front. Physiol..

[16] Waist Circumference and Waist-Hip Ratio: Report of a WHO Expert Consultation. https://www.who.int/publications/i/item/9789241501491.

[17] Gibson A.L., Wagner D.R., Heyward V.H. (2025). Advanced Fitness Assessment and Exercise Prescription.

[18] Balke B., Ware R. (1959). An experimental study of physical fitness of Air Force personnel. U. S. Armed Forces Med. J..

[19] Wasserman K., Whipp B.J., Koyl S.N., Beaver W.L. (1973). Anaerobic threshold and respiratory gas exchange during exercise. J. Appl. Physiol..

[20] Reinhard U., Müller P.H., Schmülling R.M. (1979). Determination of Anaerobic Threshold by the Ventilation Equivalent in Normal Individuals. Respiration.

[21] Matsumura N., Nishijima H., Kojima S., Hashimoto F., Minami M., Yasuda H. (1983). Determination of anaerobic threshold for assessment of functional state in patients with chronic heart failure. Circulation.

[22] Lakens D. (2013). Calculating and reporting effect sizes to facilitate cumulative science: A practical primer for t-tests and ANOVAs. Front. Psychol..

[23] Hosseini Moshkenani F., Abedi S., Shabkhiz F., Soori R., Esmaeil N. (2026). High intensity functional training versus traditional resistance training effects on inflammatory, metabolic, and physical outcomes in overweight men a randomized controlled trial. Sci. Rep..

[24] Meyer T., Lucía A., Earnest C.P., Kindermann W. (2005). A Conceptual Framework for Performance Diagnosis and Training Prescription from Submaximal Gas Exchange Parameters—Theory and Application. Int. J. Sports Med..

[25] Joyner M.J., Coyle E.F. (2008). Endurance exercise performance: The physiology of champions. J. Physiol..

[26] Scoubeau C., Carpentier J., Baudry S., Faoro V., Klass M. (2023). Body composition, cardiorespiratory fitness, and neuromuscular adaptations induced by a home-based whole-body high intensity interval training. J. Exerc. Sci. Fit..

[27] Falbová D., Sulis S., Oravská P., Hozaková A., Švábová P., Beňuš R., Vorobeľová L. (2025). The Prevalence of Normal Weight Obesity in Slovak Young Adults and Its Relationship with Body Composition and Lifestyle Habits. Bratisl. Med. J..

[28] Wrench E., Rattley K., Lambert J.E., Killick R., Hayes L.D., Lauder R.M., Gaffney C.J. (2022). There is no dose–response relationship between the amount of exercise and improvement in HbA1c in interventions over 12 weeks in patients with type 2 diabetes: A meta-analysis and meta-regression. Acta Diabetol..

[29] Lu Y., Baker J.S., Ying S., Lu Y. (2025). Effects of practical models of low-volume high-intensity interval training on glycemic control and insulin resistance in adults: A systematic review and meta-analysis of randomized controlled studies. Front. Endocrinol..

[30] Jelleyman C., Yates T., O’Donovan G., Gray L.J., King J.A., Khunti K., Davies M.J. (2015). The effects of high-intensity interval training on glucose regulation and insulin resistance: A meta-analysis. Obes. Rev..

[31] Su L., Pan Y., Chen H. (2022). The Harm of Metabolically Healthy Obese and the Effect of Exercise on Their Health Promotion. Front. Physiol..

[32] De Paoli M., Zakharia A., Werstuck G.H. (2021). The Role of Estrogen in Insulin Resistance. Am. J. Pathol..

[33] Fanghänel G., Sánchez-Reyes L., Félix-García L., Violante-Ortiz R., Campos-Franco E., Alcocer L.A. (2011). Impact of waist circumference reduction on cardiovascular risk in treated obese subjects. Cir. Cir..

[34] Kodama S. (2009). Cardiorespiratory Fitness as a Quantitative Predictor of All-Cause Mortality and Cardiovascular Events in Healthy Men and Women: A Meta-analysis. JAMA.

[35] Singh B., Cadenas-Sanchez C., Da Costa B.G.G., Castro-Piñero J., Chaput J.P., Cuenca-García M., Maher C., Marín-Jiménez N., McGrath R., Molina-García P. (2025). Comparison of objectively measured and estimated cardiorespiratory fitness to predict all-cause and cardiovascular disease mortality in adults: A systematic review and meta-analysis of 42 studies representing 35 cohorts and 3.8 million observations. J. Sport. Health Sci..

[36] Byrne N.M., Hills A.P., Hunter G.R., Weinsier R.L., Schutz Y. (2005). Metabolic equivalent: One size does not fit all. J. Appl. Physiol..

[37] García-Hermoso A., Cavero-Redondo I., Ramírez-Vélez R., Ruiz J.R., Ortega F.B., Lee D.C., Martínez-Vizcaíno V. (2018). Muscular Strength as a Predictor of All-Cause Mortality in an Apparently Healthy Population: A Systematic Review and Meta-Analysis of Data from Approximately 2 Million Men and Women. Arch. Phys. Med. Rehabil..

